# Interrater reliability of the DSM-5 and ICD-11 Criterion A for PTSD and complex PTSD in parents of children with autism using the Life Events Checklist

**DOI:** 10.1192/bjo.2024.848

**Published:** 2025-02-21

**Authors:** Kylie Hinde, Gert Martin Hald, David Hallford, Theis Lange, Mikkel Christoffer Berg B. Arendt, Silvia Pavan, David Austin

**Affiliations:** Faculty of Health, Deakin University, Melbourne, Australia; Department of Public Health, University of Copenhagen, Copenhagen, Denmark; Aarhus University, Aarhus, Denmark

**Keywords:** Autism spectrum disorders, mental health services, neurodevelopmental disorders, rating scales, trauma and stressor-related disorders

## Abstract

**Background:**

Parents of children with autism^a^ demonstrate elevated traumatic stress symptoms, but seldom receive diagnoses of post-traumatic stress disorder (PTSD) or complex PTSD. An accurate assessment of Criterion A is essential for a valid diagnosis of these disorders, yet it is uncertain whether Criterion A, as defined by the two primary international diagnostic systems (DSM-5-TR and ICD-11), yields consistent interrater reliability, when psychologists rely solely on self-report from these parents for assessing PTSD or complex PTSD.

**Aims:**

This study aims to investigate interrater reliability across psychologists when assessing Criterion A events against the ICD-11 and DSM-5-TR.

**Method:**

Ten Australian psychologists rated parents’ self-reported traumatic events related to parenting, using the Life Events Checklist for DSM-5-TR and ICD-11 Criterion A. Data from 200 randomly selected parents of children, all meeting symptom thresholds for PTSD or complex PTSD, were analysed. Bootstrapping calculated kappa coefficients, differences between ICD-11 and DSM-5-TR criteria, and self-reports of threat/no threat, with 95% confidence intervals for these differences.

**Results:**

Interrater reliability varied from poor to moderate. The ICD-11 had significantly higher reliability than the DSM-5-TR for Criterion A (*κ*_difference_ = 0.105, 95% CI 0.052–0.153, *P* < 0.001). The interrater reliability was lower when parents reported life threat, serious injury or death (*κ*_difference_ = 0.096, 95% CI 0.019–0.176, *P* = 0.007).

**Conclusions:**

This study highlights challenges in assessing PTSD and complex PTSD Criterion A in parents of children with autism, using DSM-5-TR and ICD-11 criteria with the Life Events Checklist, revealing less than adequate interrater reliability.

Post-traumatic stress disorder (PTSD) and complex PTSD are distinctive psychiatric disorders that both of the major diagnostic classification systems (DSM-5-TR^1^ and ICD-11)^2^ demand the occurrence of a specifically defined traumatic event(s) for diagnosis to be made (Criterion A). Essentially, Criterion A is sentinel; without it, no diagnosis is possible, regardless of subsequent criteria or symptom types.

## Criticism of Criterion A

However, Criterion A has been subject to substantial criticism and debate. The need to further examine issues related to Criterion A is critical, particularly in populations like parents of children with autism, who often report traumatic stress symptoms following experiences that do not meet traditional Criterion A definitions. These events may include chronic meltdowns, self-injury or persistent negative social judgement. Although these events are distressing and can lead to symptoms akin to PTSD, they are frequently excluded from a formal diagnosis because they do not align with the more narrowly defined traumatic events outlined in the DSM-5-TR or ICD-11.

Research has shown that the assessment of self-reported traumatic events in such populations can often reveal clinically significant PTSD symptoms, despite the absence of a recognised Criterion A event.^3–5^ In a recent systematic review and meta-analysis involving seven studies and 1717 participants, 24% of parents of children with autism exhibited clinically significant PTSD symptoms.^6^ These findings highlight the limitations of Criterion A in capturing the full scope of traumatic experiences in this group. This gap in the diagnostic criteria underscores the need for further research on how Criterion A applies, or fails to apply, to populations such as parents of children with autism. This study aims to investigate how Criterion A might apply or fail to apply to this parenting population.

## Criterion A in DSM-5-TR and ICD-11

For PTSD, the DSM-5-TR diagnostic criteria comprise four core symptom clusters: re-experiencing, avoidance, negative alterations in cognition and mood, and hyperarousal. In contrast, the ICD-11 comprises three clusters: re-experiencing, avoidance and persistent perceptions of heightened current threat. In the ICD-11, complex PTSD Criterion A is expanded compared with PTSD, and comprises the core PTSD symptom clusters with the addition of three disturbance in self-organisation symptom clusters: affective dysregulation, negative self-concept and disturbances in relationships.^2^ For both PTSD and complex PTSD, symptoms must be present for at least a month (DSM-5-TR PTSD) or lasting for at least several weeks (ICD-11 PTSD or complex PTSD), and impairment in daily functioning must be present.

Both diagnostic systems define Criterion A differently, leading to variations in the classification of potentially traumatic events.^7^ The DSM-5-TR restricts Criterion A to ‘exposure to death, injury, or sexual violence’,^1^ whereas the ICD-11 adopts a broader definition, requiring exposure to ‘threatening or horrific events’.^2^ Complex PTSD primarily involves ‘prolonged, or repetitive events from which escape is difficult or impossible’. These discrepancies often exclude individuals, such as parents of children with autism, whose traumatic experiences do not fit neatly within these definitions.

## Parenting of children with autism and Criterion A

A case in point is parents of children with autism, who self-report experiencing parenting-related traumatic events that do not typically meet Criterion A, such as chronic meltdowns, self-injury and negative social judgement. Yet these experiences may lead to clinically significant traumatic stress symptoms.^3–5^ Research indicates that parents of children with autism experience significantly higher levels of stress, anxiety and depressive symptoms compared with parents of typically developing children.^3,8^ A meta-analysis by Hayes and Watson,^8^ encompassing 15 studies, revealed a large effect size indicating significantly higher stress levels in parents of children with autism. Schnabel et al’s^3^ meta-analysis of 31 studies revealed significantly higher rates of depression and anxiety disorders in parents of children with autism compared with the general population. Combined, these findings raise important questions about the factors contributing to elevated psychopathological conditions in parents raising children with autism.

Autism, or autism spectrum disorder, is a complex neurodevelopmental condition characterised by differences in social communication, behaviour and sensory processing.^1,2^ Many children with autism also have co-occurring conditions, including intellectual disabilities^9^ and psychiatric disorders such as attention-deficit hyperactivity disorder, anxiety, depression and eating disorders.^10,11^ The complex, lifelong nature of caregiving, societal misconceptions and the need to navigate various systems contribute to parental stress,^12,13^ increasing the risk of parenting-related traumatic stress symptoms.

Although parenting-related events may be subjectively acknowledged as traumatic in parents of children with autism, they are typically excluded from consideration in the clinical operationalisation of Criterion A. This, therefore, limits the extent to which researchers and clinicians can use trauma-based clinical conceptualisations to understand and respond to the unique experiences of this parent population. Consequently, the assumption that Criterion A is a reliable threshold for diagnosing PTSD or complex PTSD^14,15^ may have implications for research on parents of children with autism and their treatment in clinical settings. These parents may often experience traumatic stress symptoms, but are rarely diagnosed with PTSD or complex PTSD, limiting access to trauma-informed support.^3^ Parents typically receive anxiety and mood disorder diagnoses, which may only address part of their symptoms, leading to inadequate treatment outcomes.^3^

## Criterion A and interrater reliability

PTSD Criterion A’s validity and reliability have been repeatedly questioned over decades.^7,14–20^ Criticisms include the lack of a standardised Criterion A between the DSM-5-TR and ICD-11, the limited scope of included traumatic events^17,21^ and inconsistent clinical interpretations.^18^ Studies evaluating Criterion A’s reliability consistently demonstrate difficulties in reaching consensus on what events meet the criterion.^18,19,22^ Rubin et al^18^ examined the interrater reliability (IRR) of the DSM-5-TR PTSD Criterion A, using self-report trauma descriptions from 400 university students. Their results indicated fair to moderate IRR, highlighting concerns about Criterion A’s narrow definition.

The type of traumatic event and perceived level of threat may impact IRR. Rubin et al^18^ found higher agreement on Criterion A when physical or sexual assault was endorsed, likely because of the greater likelihood of meeting the DSM criteria for death, serious injury or sexual violation.^23^ Conversely, events like natural disasters or accidents with varying threat perceptions may lead to lower rater agreement. The Life Events Checklist for DSM-5 (LEC-5) assesses perceived threat, suggesting that self-reported events with high threat may result in higher rater agreement, although this remains unexplored in the existing literature.

The Rubin et al^18^ study assessed traumatic events through self-report written descriptions instead of the standard clinical interview approach, which is known to yield higher IRR.^24,25^ Despite the advantages of clinical interviews in determining Criterion A, investigating the IRR of written self-report trauma descriptions is crucial, considering factors such as the impact of the COVID-19 pandemic on increased online PTSD/complex PTSD assessments,^18,26^ the common use of trauma event checklist questionnaires and the reliance on self-report questionnaires by primary practitioners because of time constraints.^27^

To our knowledge, Rubin et al^18^ is the only study that has investigated the IRR of the DSM-5-TR Criterion A exclusively within the context of self-report questionnaire data. No study has evaluated the IRR of PTSD Criterion A for both DSM-5-TR and ICD-11 systems in self-report data. The IRR may vary between the two systems because of differences in Criterion A definitions. Furthermore, a recent study examining the confirmatory factor analysis of the ICD-11 and DSM-5 found that the ICD-11 may provide a more valid PTSD and complex PTSD measure specifically for parents of children with autism than the DSM-5.^28^ This underscores the importance of examining Criterion A’s applicability in this population.

## The current study

The aim of this study was to examine the IRR within a group of ten independent psychologists when assessing the fit of the DSM-5-TR, ICD-11 PTSD and ICD-11 complex PTSD Criterion A for self-reported parenting-related trauma event descriptions. Based on recent findings indicating that the ICD-11 may provide a more reliable operationalisation of PTSD and complex PTSD than the DSM-5-TR,^28^ we hypothesised that the IRR for events that met Criterion A would be greater for the ICD-11 system, compared with the DSM-5-TR. Further, based on the Rubin et al study^18^ indicating that the IRR may differ based on the type of traumatic event, we hypothesised that parenting-related trauma descriptions in which serious life threat and/or serious injury or death were reported, would have a greater IRR than those descriptions that were not accompanied by a report of serious life threat and/or serious injury or death.

## Method

### Procedure

**Fig. 1:**
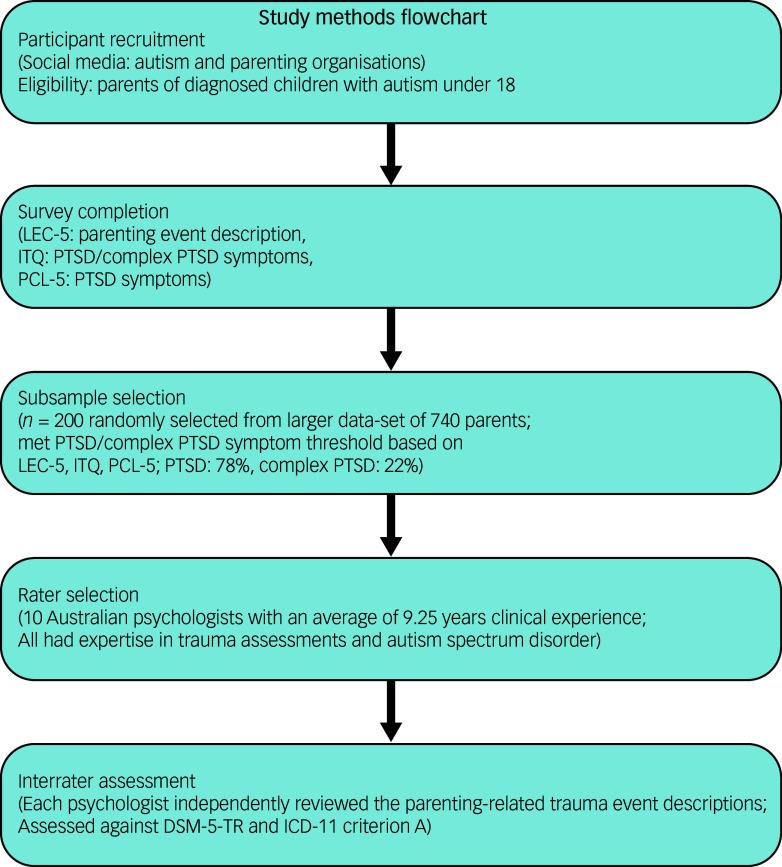
Study methods flowchart. ITQ, International Trauma Questionnaire; LEC-5, Life Events Checklist for DSM-5; PCL-5, PTSD Checklist for DSM-5; PTSD, post-traumatic stress disorder.

The present study was part of a broader cross-sectional survey study examining traumatic stress, PTSD and complex PTSD in parents of children with autism in Australia. The survey was administered with Qualtrics XM (for Mac iOS, Qualtrics International Inc., Provo, Utah, USA; www.qualtrics.com), an online survey platform, and all participants gave written informed consent by clicking ‘yes’ at the commencement of the survey. Participants were recruited via social media platforms (e.g. Facebook), targeting autism and parenting organisations. Participation in the survey was voluntary and all participants were given the opportunity to enter into a draw to win one of four headphone sets. We assert that all procedures contributing to this work comply with the ethical standards of the relevant national and institutional committees on human experimentation and with the Helsinki Declaration of 1975, as revised in 2013. All procedures involving human patients were approved by Deakin University’s Human Research Ethics Committee (approval number 2022-287) before it was preregistered on the Open Science Framework (https://osf.io/6tvng), and data collection ran from 10 March to 15 June 2023. Figure 1 presents the study methods.

### Participants

#### Raters

Psychologists renowned for their expertise in trauma and neurodiversity were recruited via K.H.’s professional network and personal invitations. Informed consent was obtained from all participating psychologists. All raters had clinical experience conducting psychological trauma assessments and working with individuals with autism and their families. All raters identified as female, averaged 43.6 years in age (s.d. = 9.56, range 24–58 years) and had an average of 9.25 years of professional experience (s.d. = 8.45, range 0.5–27 years). Nine raters (90%) completed their psychology training in Australia, and one in India. All psychologists resided and practiced in Australia and were registered as psychologists with the Australian Health Practitioner Regulation Agency.

#### Parent participants

To be included in the study, participants had to be residing in Australia and a self-reported parent of a child <18 years of age with a formal diagnosis of autism spectrum disorder. Participants first completed part two of the LEC-5, in which they were asked to report their most challenging event in parenting their child with autism (traumatic event) and to answer the additional questions relating to this event, which were as follows: ‘When did the event(s) occur?’, ‘How was it experienced?’, ‘Did the event involve actual or threatened death or serious injury?’ and ‘Was the event repeated and how many times?’. The participants were then asked to complete the International Trauma Questionnaire (ITQ) and the PTSD Checklist for DSM-5 (PCL-5). A total of 740 participants completed the survey, with 509 identifying as parents of a child with autism, and the remaining 231 were parents who identified that their children did not have an autism, nor any, clinical diagnosis. A subsample (*n* = 200) of parents of children with autism that met the PTSD (*n* = 156, 78%) or complex PTSD (*n* = 44, 22%) symptom threshold on the PCL-5 and/or the ITQ were then randomly selected for inclusion in the present study (see Table 1). This sample size was chosen to balance the capacity to detect meaningful effects with practical constraints like rater time and availability. Among the sample of 200 parents, 94 (47%) met the PTSD symptom inclusion criteria and 106 (53%) met the ICD-11 complex PTSD criteria. Participants who met the symptom inclusion criteria for both PTSD and complex PTSD were counted as complex PTSD only. Of the 94 parents who met the PTSD symptom inclusion criteria, 34 (64.5%) of these parents satisfied both the DSM-5 PTSD and ICD-11 PTSD criteria, 45 (22.5%) met only the DSM-5 PTSD symptom criteria and 15 (7.5%) met solely the ICD-11 criteria. Both the ITQ and the PCL-5 assess trauma symptoms regardless of Criterion A presence.

**Table 1:** Counts of Life Events Checklist for DSM-5 (LEC-5) items, as reported by parent participants (*n* = 200)

Life events	Number of times endorsed	%
Events reported that *did* involve: Life threat, serious injury and/or death	81	40.5
Events reported that *did not* involve: Life threat, serious injury and/or death	119	59.5
How the parent reported experiencing the event:^a^		
It happened to me directly	106	53.0
I witnessed it	86	43.0
I learned about it happening to my child	39	19.5
I was repeatedly exposed to details about it as part of my parenting role	66	33.0
Events reported as having occurred more than once	151	75.5
Events that were reported as being both repeated and involving:		
Life threat, serious injury and/or death	59	29.5

a. This item allowed participants to select more than one way they experienced the event; percentage was calculated from the number of total events rated (*n* = 200).

### Measures

#### ITQ and PCL-5

Study participants completed the PCL-5 scale^29^ and the ITQ^30^ to assess PTSD (PCL-5 and ITQ) and complex PTSD symptoms (ITQ only). Both scales demonstrate robust psychometric properties (PCL-5, *α* = 0.94;^31^ ITQ, *α* = 0.79^30^). For both PCL-5 PTSD (DSM-5-TR) and ITQ PTSD and complex PTSD (ICD-11), clinically significant symptoms were determined by scoring ≤2 on an endorsed symptom, followed by the diagnostic rule of each system. DSM-5-TR PTSD requires at least one endorsed symptom for Criterions B and C and two for Criterions D and E. ICD-11 PTSD necessitates at least one endorsed symptom for re-experiencing, avoidance, sense of threat and functional impairment symptom clusters. ICD-11 complex PTSD requires the PTSD criteria to be met in addition to endorsing at least one symptom for affective dysregulation, negative self-concept, disturbances in relationships and self-organisation impairment symptom clusters. If a participant met symptom criteria for both PTSD and complex PTSD, they were counted as complex PTSD only. In this study, the PCL-5 demonstrated acceptable reliability (*α* = 0.79) and the ITQ exhibited excellent reliability (*α* = 0.94).

#### LEC-5 part two

The LEC-5 was used to assess participants’ exposure to potentially traumatic parenting-related experiences.^29^ The LEC-5 measure was modified to be more applicable to parenting-related stressful experiences by changing the instruction from ‘briefly describe the worst event’ to ‘briefly describe the worst event in parenting your autistic child’. The modified LEC-5 version is available in the Supplementary Material. The LEC-5 used in this study included six of the original eight checklist items asking participants to describe their worst parenting event/s, how it was experienced, if it involved actual or threatened death and/or serious injury, and the frequency of exposure. The question assessing parenting-related sexual violence was removed because of the scale’s lack of relevance for parents of children with autism and the absence of research evidence of sexual violence of children with autism toward their parents, thereby streamlining our survey to minimise participant burden and focus on key variables of interest. The question about the timing of the event was administered to participants, but not shared with rating psychologists, as it was unnecessary for assessing DSM-5 or ICD-11 Criterion A. In this study, Cronbach’s alpha for the LEC-5 was excellent (Cronbach’s *α* = 0.94).

The psychologists assessed 200 individual parent descriptions for meeting Criterion A for PTSD according to the DSM-5-TR and ICD-11, and complex PTSD according to the ICD-11. Table 1 presents the total count and percentage of reported LEC-5 items by parent participants.

### Data analysis

No psychologist demographic data or LEC-5 event rating data was missing. For participants, no LEC-5 event data was missing and <2.5% of demographic data was missing at random, which was considered a relatively low amount.^32^ Since participant demographic data was unrelated to key study outcomes, missing demographic data was reported in Table 2 without further action.

**Table 2:** Sociodemographic characteristics of the parent sample (*n* = 200)

	Parents of children with autism				
Characteristics	Mean	s.d.	Range	*n*	%
Parent age (years)	41.9	6.9	21–58	199	0.5 (missing data)
Child age (years)	10.8	4.2	2–17	200	
Parent gender					
Female				165	82.5
Male				32	14.3
Other				3	1.5
Missing data				1	0.5
Child gender					
Female				85	42.5
Male				110	55
Other				5	2.5
Parent relationship to child					
Mother				165	82.5
Father				32	14.3
Foster parent/other				3	1.5
Missing data				1	0.5
Ethnicity					
Caucasian or White European				165	82.
Asian				14	7
Other				20	10
Missing data				1	0.5
Highest education					
University degree or higher				103	51.5
Occupational/technical/vocational training				63	31.5
High school				27	13.5
Other				4	2
Missing data				3	2.5
Current employment					
Full time				58	29.0
Part time				61	30.5
Stay-at-home parent				58	29
Unemployed and other				20	10
Missing data				3	1.5

#### IRR

The Python Language Reference (version 3.11.4 for Mac iOS; Python Software Foundation, Beaverton, Oregon, USA) was used to conduct statistical analyses. Python is a cross-platform programming language and can be downloaded from https://www.python.org/downloads/. Given the ten raters, the use of categorical data and the emphasis on assessing interrater agreement when considering chance agreement, Fleiss’s kappa was considered the most suitable statistic for addressing the research question.^33^ Fleiss’s kappa assesses the level of agreement among more than two raters, by comparing categorical ratings for a set of items and taking into account both the observed agreement among the raters, and the agreement expected by chance. Interpretation of the kappa values was based on Fleiss’s guidelines: ≤0.20 = poor agreement, 0.21–0.40 = fair agreement, 0.41–0.60 = moderate agreement, 0.61–0.80 = substantial agreement, 0.81–1.00 = almost perfect agreement.^33^ Similar to the methodology of Ruben et al,^18^ we also calculated Light’s kappa as a measure of confidence in the robustness of our agreement assessment among all rater pairs.^34^ Light’s kappa is the mean of kappas computed from all possible pairs of raters, with a range interpreted as, for example, 1 indicating perfect agreement among all pairs.^34^

To estimate the sampling distribution and to be able to make inferences about the wider population, bootstrapping was used to test the two hypotheses. The first hypothesis posited that the IRR for Criterion A would be greater for the ICD-11 system compared with the DSM-5-TR. The second hypothesis proposed that descriptions of parenting-related trauma involving a serious life threat and/or serious injury or death would exhibit a greater IRR compared with those lacking such severity indicators. To address these hypotheses, we generated 1500 resampled data-sets with replacements, simulating variations in our original data and calculated the confidence intervals, using the 2.5th and 97.5th percentiles of the kappa distribution(s). Differences were calculated by subtracting the difference between IRR values and then calculating subsequent 95% confidence intervals for that difference. Confidence intervals that did not cross zero were considered significance at *P* < 0.050. IRR was calculated for the original sample without bootstrapping, and can be viewed in Supplementary Table 1.

## Results

Overall, for PTSD across both ICD-11 and DSM-5-TR Criterion A, a moderate IRR was found (*κ* = 0.427, 95% CI 0.426–0.428). Supporting our first hypothesis, IRR among ten raters assessing 200 parent descriptions of their worst parenting experience, showed significantly higher IRR for ICD-11 PTSD Criterion A compared with DSM-5-TR PTSD Criterion A (*κ*_difference_ = 0.105, 95% CI 0.052–0.153, *P* < 0.001).

For our second hypothesis, in which we predicted descriptions of parenting-related trauma involving serious threats to life, serious injury or death would exhibit higher IRR compared with descriptions without such qualifiers, we observed that the IRR decreased to a fair level of agreement when parents endorsed life threat, serious injury or death. Contrary to our hypothesis, across both the ICD-11 and DSM-5, the IRR was significantly lower (*κ*_difference_ = 0.103, 95% CI 0.019–0.176, *P* = 0.007) when these qualifiers were endorsed compared with when they were not endorsed. For complex PTSD Criterion A (ICD-11), IRR was poor for events involving life threat, serious injury or death, and fair otherwise. Table 3 presents the IRR coefficients and their respective confidence intervals across the raters and the ICD-11 and DSM-5-TR Criterion A. Light’s kappa coefficient was comparable to Fleiss’s kappa coefficient, with the largest difference being 0.045, indicating that the level of agreement measured was consistent and not heavily influenced by the choice of the agreement statistic.

**Table 3:** Interrater reliability across ten independent psychologists and the ICD-11 and DSM-5-TR Criterion A – bootstrapped data^a^

Condition	DSM-5-TR PTSD (*κ*)	95% CI	ICD-11 PTSD (*κ*)	95% CI	ICD-11 complex PTSD (*κ*)	95% CI (complex PTSD)
Overall Criterion A	0.390	[0.336–0.444]	0.495	[0.436–0.555]		
Life threat reported	0.120	[0.047–0.202]	0.261	[0.166–0.364]	0.174	[0.069–0.278]
Non-life threat reported	0.420	[0.337–0.449]	0.523	[0.440–0.601]	0.297	[0.228–0.366]

*κ* indicates Fleiss’s kappa; life threat reported includes serious injury and/or death. PTSD, post-traumatic stress disorder.

a. This table provides the interrater reliability values for DSM-5-TR and ICD-11 PTSD Criterion A, as well as ICD-11 complex PTSD. Hypothesis 1 predicts that the interrater reliability for ICD-11 PTSD Criterion A would be higher than for DSM-5-TR PTSD Criterion A, and hypothesis 2 predicts higher interrater reliability for descriptions involving serious life threat, injury or death (life threat reported) compared with non-life threat descriptions. The main comparisons and *κ*_difference_ values for these hypotheses are reported in the text.

Out of 200 events, 81 were endorsed as a Criterion A event by the parent. However, of these, only five (2.5%) reached full rater consensus for meeting DSM-5-TR PTSD Criterion A (involving exposure to death, threatened death, actual or threatened serious injury, or actual or threatened sexual violence), 15 (7.5%) met ICD-11 PTSD Criterion A (involving exposure to an event or situation of an extremely threatening or horrific nature, either short- or long-lasting) and none met ICD-11 complex PTSD Criterion A (involving exposure to an event or series of events of an extremely threatening or horrific nature, most commonly prolonged or repetitive events from which escape is difficult or not possible).

Additional analyses, reviewing the raters’ years of experience, were conducted. We found significant differences in IRR for ICD-11 PTSD and overall IRR between experienced psychologists (≥10 years) and early career psychologists (0–3 years) (ICD-11 PTSD: *κ*_difference_ = 0.242, 95% CI 0.110–0.373, *P* ≤ 0.001; overall IRR: *κ*_difference_ = 0.119, 95% CI 0.034–0.207, *P* = 0.002). Early career psychologists demonstrated significantly higher IRR than experienced psychologists for DSM-5 PTSD (*κ*_difference_ = 0.119, 95% CI 0.034–0.207, *P* < 0.001). Both mid-career and experienced psychologists demonstrated significantly higher IRR than early career psychologists for ICD-11 complex PTSD (mid-career: *κ*_difference_ = 0.209, 95% CI 0.105–0.306, *P* < 0.001; experienced: *κ*_difference_ = 0.276, 95% CI 0.170–0.384, *P* < 0.001).

## Discussion

The aim of this study was to examine psychologists’ IRR in assessing parenting-related events for meeting PTSD (DSM-5-TR and ICD-11) and complex PTSD (ICD-11) Criterion A in a cohort of parents with children with autism. The results indicated that the reliability of both diagnostic systems as measured by the LEC-5, ranged from poor to moderate, falling below what is typically considered acceptable.^35^ This outcome is concerning for two main reasons. First, the threshold role Criterion A plays in the diagnostic process for both PTSD and complex PTSD, and second, that psychologists, who are often responsible for assessing and diagnosing PTSD and complex PTSD, had such low agreement in assessing Criterion A in this parent population. Furthermore, our study deliberately included only parents that met PTSD or complex PTSD symptom thresholds, which heightens the significance of these findings. We propose there may be several reasons for these findings.

### Alignment with Criterion A

Our low IRR finding is likely the result of parent-reported traumatic events not clearly aligning with ICD-11 and DSM-5-TR Criterion A descriptions. Raters were specifically asked to refer to the ICD-11 and DSM-5-TR criterion A definitions when assessing these parent-reported events. Many descriptions involved themes of child suicidality and self-harm, aggression toward others, bullying/victimisation and life-threatening behaviours such as elopement. These themes are typically acknowledged as traumatic events in clinical and research contexts.^36,37^ However, in this study, these events showed low IRR in meeting Criterion A, even when parents endorsed them as involving life threat, serious injury or death. Parent reports generally fell into two categories: potentially traumatic events (e.g. bullying, elopement and meltdowns involving aggression) and unequivocally traumatic events (e.g. suicide attempts or aggression causing serious injury). The distinction between potential and unequivocal traumatic events became challenging when parent-reported events aligned with Criterion A examples but lacked endorsement for life threat, serious injury or death, or were not clearly consistent with Criterion A but were endorsed as involving life threat, serious injury or death. In such cases, raters faced the decision of prioritising alignment with Criterion A or alignment with the parent’s report of life threat, serious injury or death.

### Endorsement of threat

Contrary to expectations, trauma descriptions in which parents indicated serious life threat and/or serious injury or death had lower IRR compared with descriptions without these qualifiers. Among the 81 events reported as life-threatening or involving serious injury or death, only 20 achieved complete consensus as meeting Criterion A. It is possible that raters focused more on event characteristics than the perceived level of threat, which was unexpected, as it was assumed that raters would typically endorse Criterion A when parents reported such threats, regardless of the event type. There may be several possible explanations for this outcome.

First, some parents may have perceived events as more ‘serious’ or ‘life-threatening’ than they objectively were, leading to overreporting threat. Psychologists, as a result of their training, may have approached the assessment of parent-reported events with a more impartial perspective when determining the level of threat. A second explanation is that certain aspects of parent descriptions led raters to minimise or normalise reported threat. This could suggest limitations in psychologists’ understanding and expertise in assessing PTSD and complex PTSD Criterion A as per the DSM-5-TR and ICD-11 in parents of children with autism. It may be possible that psychologists were impacted by diagnostic overshadowing bias,^38^ where they misattributed symptoms to existing conditions, such as assuming that parents of children with autism experience caregiving stress and challenges as a normative part of their role, leading to an underestimation of the impact of traumatic experiences of these parents.

Although it is well established that parents of children with autism experience high rates of anxiety, depression and stress-related conditions,^3^ the recognition of traumatic stress-related disorders (e.g. PTSD and complex PTSD) in these parents is limited. This may lead psychologists to attribute trauma-based symptoms to more conventional conditions like anxiety, depression and stress, indicating diagnostic overshadowing bias. This bias highlights the challenges in diagnosing mental health conditions in unconventional populations or experiences that do not neatly fit into conventional diagnostic criteria.

### ICD-11 and DSM-5-TR Criterion A

The ICD-11 showed significantly higher agreement for PTSD Criterion A than the DSM-5-TR, with complex PTSD IRR ranging from low (when life threat, serious injury or death was endorsed) to fair (without such endorsement). Among the 20 events that achieved perfect IRR for meeting PTSD Criterion A, 15 met the ICD-11 Criterion A, whereas only five met the DSM-5-TR Criterion A. This outcome may be attributed to the DSM-5-TR Criterion A definition being more restrictive than its ICD-11 equivalent.^15^ These findings are consistent with previous research,^15^ and suggest that ICD-11 PTSD and complex PTSD Criterion A may better encompass experiences that may be excluded through application of DSM-5 diagnostic criteria.

Qualitative research on parents of children with autism often describes parenting-related experiences as ‘horrifying’, ‘devastating’ and ‘threatening’.^36,39^ Similar descriptors also appeared in this study, aligning with ICD-11 Criterion A, which involves exposure to extremely threatening or horrific events. However, chronic meltdowns, elopement and sensory-seeking behaviours like faecal smearing and self-biting, showed low interrater agreement for meeting Criterion A in this study. This is consistent with previous studies,^3,4,36^ which seldom recognised these types of parenting-related events as Criterion A events.

Examples in both the ICD-11’s complex PTSD Criterion A (torture, concentration camps, slavery etc.) and DSM-5-TR’s PTSD Criterion A (e.g. death, injury, sexual violence, etc.) do not typically apply to this parent population. This lack of clear application to parenting events affects raters’ ability to assess if such events meet Criterion A. The examples aim to guide clinicians, but can impede reliability for some populations. In this study, non-alignment with ICD/DSM examples negatively affected the IRR, indicating variability in Criterion A ratings resulting from a reliance on individual judgements in the absence of clear guidelines.

Overall, higher agreement was observed for events not meeting Criterion A compared with those that did. Among the 200 parent-reported traumatic events, 151 occurred multiple times and 59 (29.5%) involved parent reports of life threat, serious injury or death. Complex PTSD Criterion A relates to interpersonal, threatening and entrapping experiences, consistent with some parent descriptions in existing literature, such as pervasive public meltdowns, self-harming, suicide attempts, aggressive behaviour toward family members, injuries and elopement in high-risk places.^4^

Inconsistent interpretation of Criterion A may result from unclear definitions, as seen in complex PTSD Criterion A (ICD-11) lacking clarity on terms like ‘prolonged and event repetition frequency’. This ambiguity forces reliance on clinical judgement, introducing inconsistency. In this study, descriptions involving child-related suicidality, self-harm, disordered eating and aggression had low IRR for meeting complex PTSD Criterion A. Developing clearer definitions and guidelines for terms like ‘prolonged’ and ‘repeats of an event or series of events’, along with specific time frames or frequency thresholds, may enhance consistency and certainty for clinicians when assessing against complex PTSD Criterion A.

In contrast to Rubin et al,^18^ where graduate students showed greater agreement than experienced raters, our study reveals variable IRR differences between experienced psychologists and early career psychologists. Early career psychologists showed greater agreement than experienced psychologists when rating DSM-5 PTSD, whereas experienced psychologists demonstrated higher agreement in rating ICD-11 PTSD, ICD-11 complex PTSD and overall agreement.

These findings imply that recently trained Australian psychologists, more familiar with the DSM system, exhibit greater agreement in identifying PTSD symptoms according to DSM criteria. The lower use of the ICD-11 in Australian university and healthcare psychology settings may necessitate further clinical training or experience, which potentially explains why experienced clinicians had higher agreement with the ICD-11.

### Future recommendations

Our findings are broadly consistent with Rubin et al,^18^ who also reported fair to moderate IRR for Criterion A. Rubin et al recommended using self-report measures like the LEC-5 with multiple raters, suggesting comprehensive standardised rater training and group discussions to improve agreement. However, our study, involving advanced clinical psychology-trained raters, did not achieve acceptable agreement in assessing Criterion A for parents of children with autism. Specialised training focusing on Criterion A within diagnostic systems may enhance IRR, as seen in web-based programmes for PTSD assessment.^40^ To the best of our knowledge, there are currently no clinical training programs targeting the assessment of PTSD or complex PTSD specifically in parents of children with autism.

Notably, our study suggests a possible inadequacy of Criterion A in capturing the unique trauma experiences of parents of children with autism, which may contribute to low rater agreement. Existing literature proposes revising or expanding Criterion A,^7,15,21^ but modifying it raises concerns of increased false positives.^15^ Although the ICD-11’s less prescriptive Criterion A resulted in more positive cases than the DSM-5-TR, both systems captured significant PTSD or complex PTSD symptoms in parents. We suggest adopting less specific qualifying examples in diagnostic systems for parents of children with autism, to improve inclusivity and relevance, thereby facilitating more accurate trauma assessments and intervention.

### Limitations

This study focused solely on Criterion A, using a self-report measure that limited the depth of event characterisation, potentially affecting the perceived intensity of parental experiences. Including clinical interviews alongside self-report measures in future studies is recommended for more comprehensive assessment. Another limitation relates to the use of kappa statistic interpretation, which is context-dependent, considering variations in population characteristics and clinical judgement among psychologists. Caution is advised in drawing conclusions based solely on kappa statistics, considering differences in training, diagnostic experience and cultural competency.

Additionally, 82.5% of parents and all raters in the study identified as female, which may limit the statistical generalisability of the findings. This gender imbalance should be considered when interpreting the results. Finally, the exclusive focus on parents of children with autism limits the study’s external validity. Future research should replicate studies across diverse populations to validate findings and enhance generalisability.

In conclusion, to our knowledge, this study is the first to address the challenges of assessing PTSD and complex PTSD Criterion A in parents of children with autism by using the DSM-5-TR and ICD-11 as assessed by the LEC-5. Findings reveal low IRR below acceptable levels in clinical and research settings, raising concerns about accurate diagnosis when relying on self-report measures such as those used in this study. This study did not provide insights into potential challenges associated with face-to-face clinical settings, where follow-up questions determine caseness. Our findings call into question the adequacy of existing diagnostic systems for capturing the unique trauma experiences of these parents. A discrepancy between parent reports and diagnostic criteria, particularly in cases involving threats to life or severe harm, highlights the need for a more comprehensive trauma assessment approach, including specialised training for professionals assessing trauma in parents of children with autism. Future revisions or expansions of Criterion A may enhance diagnostic accuracy and improve access to appropriate interventions for parents with traumatic stress symptoms.

## Supporting information

Hinde et al. supplementary material Hinde et al. supplementary material

## Data Availability

The study pre-registration, along with the Python syntax and original and bootstrapped dataset, are openly available on the Open Science Framework (OSF) platform: (https://osf.io/6tvng/?view_only=4eb48a78189f46278f013991634601cc). Parent-reported trauma descriptions are not available to be shared due to the risk of disclosing identifying information.

## Supplementary material

The supplementary material is available online at https://doi.org/10.1192/bjo.2024.848.
