# Interrater reliability of the DSM-5 and ICD-11 Criterion A for PTSD and complex PTSD in parents of children with autism using the Life Events Checklist – ERRATUM

**DOI:** 10.1192/bjo.2025.53

**Published:** 2025-06-30

**Authors:** Kylie Hinde, Gert Martin Hald, David Hallford, Theis Lange, Mikkel Christoffer Berg B. Arendt, Silvia Pavan, David Austin

**Keywords:** Autism spectrum disorders, mental health services, neurodevelopmental disorders, rating scales, trauma and stressorrelated disorders, erratum

This article was originally published with an incorrect e-number, e34. The correct e-number is e36 and all versions of the article have been updated to include the correct e-number.
