# Supplementary material for: Interrater reliability of the DSM-5 and ICD-11 Criterion A for PTSD and complex PTSD in parents of children with autism using the Life Events Checklist
Source: BJPsych Open. 2025 Feb 21;11(2):e36. doi: 10.1192/bjo.2024.848 (PMC12001950; doi:10.1192/bjo.2024.848)
Supplement: Hinde et al. supplementary material [file S2056472424008482sup001.docx]

Supplementary Data

***Modified LEC-5 (part-two)***

These next questions ask about problems that you may have had after a very stressful parenting experience or experiences parenting your autistic child. It could be something that happened to you directly involving your child, something you witnessed of your child, or something you learned happened to your child. Please answer a few questions about your worst event in parenting your autistic child, which for this questionnaire means the event or events that currently bother you the most. It could be a single event or an event that has occurred multiple times.

**If you have more than one autistic child, please answer the following questions based on the child with the most severe challenges.**

- Briefly identify the worst event in parenting your autistic child (for example, what happened, who was involved, etc):
- How long ago did it happen (please estimate if you are not sure)
- How did you experience it?

It happened to me directly

I witnessed it

I learned about it happening to my child

I was repeatedly exposed to details about it as part of my parenting role

Other (please describe)

- Was someone’s life in danger?

Yes, my life

Yes, my child’s life

Yes, someone’s life

No

- Was someone seriously injured or killed?

Yes, I was seriously injured

Yes, my child was seriously injured or killed

Yes, someone else was seriously injured or killed

No one was seriously injured or killed

- If the event involved the death or serious injury of your child, or a close family member, or a close friend, was it due to some kind of accident or violence, or was it due to natural causes?

Accident involving my child

Violence involving my child

Natural causes

Not applicable (the event did not involve the death or serious injury of my child, a close family member or close friend)

- How many times have you experienced this event if it was repeated?

Just once

More than once (please specify or estimate the total number of times you have had this experience

Table S1.

*Inter-rater Reliability across 10 Independent Psychologists Including Categories According to Experience and 8 Conditions – Original Data*

|  | Psychologist category | | | | | | | |
| --- | --- | --- | --- | --- | --- | --- | --- | --- |
|  | Early career (*n* = 3) | | Mid-career (*n* = 3) | | Experienced (*n* = 4) | | Combined psychologists (*n* = 10) | |
| Condition | κ | 95% C1  [LL, UL] | κ | 95% C1  [LL, UL] | κ | 95% C1  [LL, UL] | κ | 95% C1  [LL, UL] |
| DSM-5-TR PTSD | .401 | [.321, .481] | .409 | [.329, .489] | .304 | [.247, .361] | .391 | [.370, .411] |
| life threat reported* | .195 | [.046, .344] | -.056 | [-.205, .093] | .013 | [-.092, .118] | .124 | [.086, .162] |
| ICD-11 PTSD | .425 | [.345, .505] | .373 | [.292, .453] | .667 | [.610, .723] | .496 | [.476, .517] |
| life threat reported* | .185 | [.036, .333] | .104 | [-.044, .253] | .471 | [.365, .576] | .268 | [.230, .306] |
| ICD-11 complex-PTSD | .019 | [-.061, .099] | .230 | [.150, .310] | .302 | [.245, .358] | .268 | [.247, .289] |
| life threat reported* | -.074 | [-.223, .074] | .218 | [.069, .366] | .208 | [.103, .313] | .184 | [.146, .223] |
| Overall | .341 | [.294, .387] | .409 | [.363, .455] | .460 | [.428, .493] | .428 | [.416, .440] |
| life threat reported* | .232 | [.146, .318] | .297 | [.211, .383] | .366 | [.305, .427] | .334 | [.311, .356] |
